# Laboratory-Enhanced Dengue Sentinel Surveillance in Colombo District, Sri Lanka: 2012-2014

**DOI:** 10.1371/journal.pntd.0004477

**Published:** 2016-02-29

**Authors:** Hasitha Tissera, Ananda Amarasinghe, Sunethra Gunasena, Aruna Dharshan DeSilva, Leong Wei Yee, October Sessions, Chanaka Muthukuda, Paba Palihawadana, Wolfgang Lohr, Peter Byass, Duane J. Gubler, Annelies Wilder-Smith

**Affiliations:** 1 Epidemiology Unit, Ministry of Health, Colombo, Sri Lanka; 2 National Dengue Control Unit, Colombo, Sri Lanka; 3 Medical Research Institute, Colombo, Sri Lanka; 4 Genetech Research Institute, Colombo, Sri Lanka; 5 Lee Kong Chian School of Medicine, Nanyang Technological University, Singapore, Singapore; 6 Program in Emerging Infectious Diseases, Duke-NUS Graduate Medical School, Singapore; 7 Department of Public Health and Clinical Medicine, Epidemiology and Global Health, Umeå University, Umeå, Sweden; Florida Department of Health, UNITED STATES

## Abstract

**Introduction:**

Dengue has emerged as a significant public health problem in Sri Lanka. Historically surveillance was passive, with mandatory dengue notifications based on clinical diagnosis with only limited laboratory confirmation. To obtain more accurate data on the disease burden of dengue, we set up a laboratory-based enhanced sentinel surveillance system in Colombo District. Here we describe the study design and report our findings of enhanced surveillance in the years 2012–2014.

**Methods:**

Three outpatient clinics and three government hospitals in Colombo District that covered most of the Colombo metropolitan area were selected for the sentinel surveillance system. Up to 60 patients per week presenting with an undifferentiated fever were enrolled. Acute blood samples from each patient were tested by dengue specific PCR, NS1 ELISA and IgM ELISA. A sub-set of samples was sent to Duke-NUS Singapore for quality assurance, virus isolation and serotyping. Trained medical research assistants used a standardized case report form to record clinical and epidemiological data. Clinical diagnoses by the clinicians-in-charge were recorded for hospitalized cases.

**Results:**

Of 3,127 febrile cases, 43.6% were PCR and/or NS1 positive for dengue. A high proportion of lab confirmed dengue was observed from inpatients (IPD) (53.9%) compared to outpatient (clinics in hospitals and general practice) (7.6%). Dengue hemorrhagic fever (DHF) was diagnosed in 11% of patients at the time of first contact, and the median day of illness at time of presentation to the sentinel sites was 4. Dengue serotype 1 was responsible for 85% of the cases and serotype 4 for 15%. The sensitivity and specificity of the clinicians’ presumptive diagnosis of dengue was 84% and 34%, respectively.

**Conclusion:**

DENV-1, and to a lesser degree DENV-4, infection were responsible for a high proportion of febrile illnesses in Colombo in the years 2012 to 2014. Clinicians’ diagnoses were associated with high sensitivity, but laboratory confirmation is required to enhance specificity.

## Author Summary

The dramatic spread of epidemic dengue underscores the urgent need for better surveillance and control of this disease. The main purpose of surveillance is to provide timely and more accurate information to institute preventive or control measures for epidemic dengue. Dengue has emerged as a major public health problem in Sri Lanka. To obtain more data on the burden of dengue, a laboratory-based enhanced sentinel surveillance system was established in metropolitan Colombo. In this paper we describe how we set up the sentinel sites and report the results of the first two years of enhanced surveillance (2012–2014). Of 3,127 patients presenting with acute onset of fever, 43.6% had laboratory confirmed dengue (PCR of NS1 positive), mainly caused by dengue serotype 1. Clinicians’ diagnoses were associated with high sensitivity, but our findings also show that laboratory confirmation is required to enhance specificity. There were 5 deaths, all due to dengue hemorrhagic fever.

## Introduction

Dengue is one of the most important vector-borne viral disease worldwide, transmitted by *Aedes* mosquitoes and caused by any of the four dengue virus serotypes [1]. The extent of dengue transmission and therefore the risk of outbreaks is determined by a combination of various factors; these include the virulence of the dominant viral genotype, the level of herd immunity, the abundance, species and vector competence of *Aedes* mosquitoes; weather and climate variables, human population density, and the distribution and movement of the viruses, vectors and humans [2]. The dramatic global spread and increased frequency and magnitude of epidemic dengue/dengue hemorrhagic fever (DEN/DHF) in the past 40 years underscores the critical need for more effective surveillance, prevention and control of this disease [3]. The main purpose of surveillance is to provide the information necessary for risk assessment, program evaluation, and to allow timely action to prevent or control epidemic dengue. Most endemic countries do not have adequate dengue surveillance and rely on passive syndromic surveillance, which is known to lack sensitivity and specificity [4]. Many experts have called for more active surveillance to provide better early warning signals to trigger effective dengue emergency response [4].

In 2011, the European Commission funded three European consortia with a specific focus on surveillance and control of dengue [5]. One of these three dengue consortia, “DengueTools” (www.denguetools.net) was funded to set up a laboratory-enhanced sentinel surveillance in Sri Lanka [6].

In Sri Lanka, dengue increasingly poses a significant socio-economic and public health burden [7]. The geographic spread, incidence and severity of disease is of major concern ever since the first dengue hemorrhagic fever epidemic occurred in 1989 [8]. Periodic epidemics have become progressively larger, peaking with the 2009–2014 epidemic with 28,000 to more than 40,000 cases reported each year (44,461 cases in 2012)[9], which may be partly due to increasing awareness and diagnosis, but is more likely a true reflection of dengue emergence in this country, as also observed in the Asia Pacific region. During that same period, the disease dramatically expanded to the whole island. Historically surveillance was passive with mandatory dengue notifications based on illness clinically compatible with dengue and only limited laboratory confirmation.

To augment the existing passive surveillance in the country, we set up a laboratory-enhanced sentinel surveillance system in Colombo District, Sri Lanka. Here we describe the study sites, research design and the findings of the first two years.

## Methods

### Ethics statement

Ethical approval for the study was obtained from the Ethics Review Committee, Faculty of Medicine, University of Colombo, Sri Lanka. All informed consent by participants were in written format. Parental consent was obtained for all participants up to the age of 18 years. For children below 12 years of age, the informed consent was written and signed by their parents/guardians while for those who were above 18 years of age, informed consent was written and signed by them. All data that were analyzed were anonymized.

### Study setting

Colombo district reports the highest dengue caseload for any given year. Colombo is one of the 26 administrative districts in Sri Lanka, located in the Western part of the country. The national population is 20.4 million (Census 2012), of which approximately 12% live in Colombo District. The greater part of the area is urban (54.6%), with the country’s highest population density of 3,438 km^-2^ (Dept. of Census and Statistics, Sri Lanka). The weather is wet (average rainfall of 2,306 mm/year) and tropical (an average temperature of 27°C). Colombo is the commercial and administrative hub of the country with an average of one million people commuting in and out of the city on a daily basis. Free health-care service is available to the entire population.

### Sentinel sites

**Fig 1:**
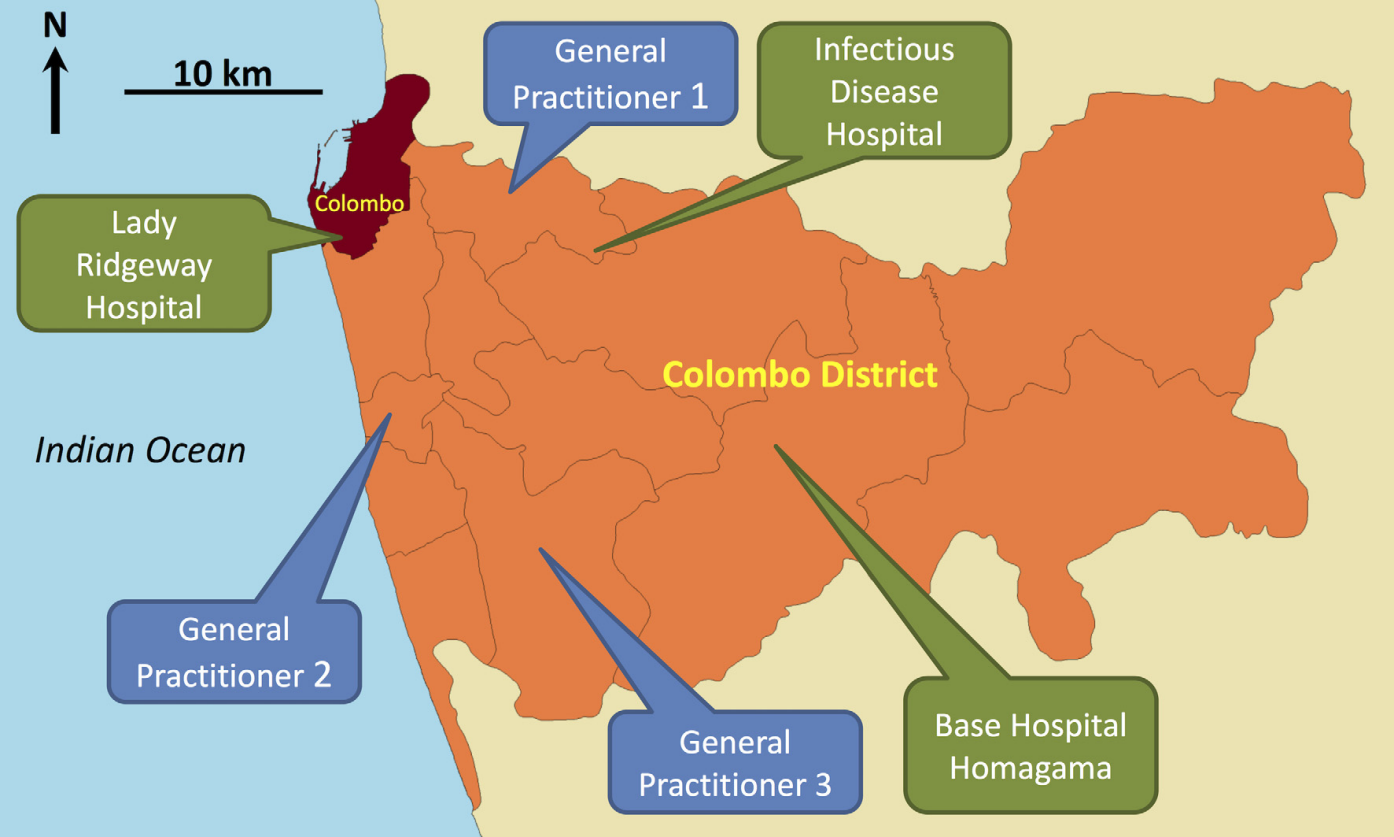
Map of Colombo District, Sri Lanka, showing the locations of six dengue fever surveillance sites.

We selected three government hospitals (Lady Ridgeway Hospital for Children (LRH), Infectious Disease Hospital (IDH), and District Base Hospital–Homagama (BHH)) and three general practitioner (GP) clinics. LRH is the national Centre of Excellence for children on tertiary medical care with over 900 beds and an outpatient attendance over one million per year. IDH is the national Centre of Excellence on infectious disease medical care. The selection of sentinel sites was made ensuring wide geographical coverage in the District (Fig 1).

### Recruitment

Patients presenting to any of the 6 sentinel sites were recruited if they fulfilled the following inclusion criteria: undifferentiated febrile illness with a duration of less than 7 days (with fever defined as temperature greater than 38.0°C) and providing informed consent. Total recruitment per week was capped at 60 patients. Cases from LRH were enrolled on a daily basis (maximum 10 per week), and from the other two hospitals once a week (for patients presenting on one specified day of the week, maximum 10 on that particular day). In addition, the research team visited the three GP clinics once per week and enrolled up to 10 febrile patients that presented with an undifferentiated fever on that day. From all enrolled patients, a 2.5–5.0 ml venous blood sample was collected and transported to the Medical Research Institute (MRI) laboratory on the same day.

### Clinical data

Interviewer administered, pre-tested case report forms (CRF) were developed to collect the patient clinical and epidemiological data. The CRFs were administered and filled in by research assistants who were pre-intern medical officers trained in the WHO dengue case classification and in obtaining data in a standardized manner. Data were collected on age, sex, residency, presumptive clinical diagnosis, signs and symptoms with duration by systems, past medical, drug and vaccination history, laboratory test report findings on day of first presentation to the sentinel site, and treatment received. Day of illness was defined as the day since onset of fever. The medical research assistants recorded their presumptive diagnosis at the time of the interview based on the presenting symptoms, as well as the presumptive diagnosis that was recorded by the clinicians-in-charge in the patients’ source records (not the CRFs) at first contact. All hospitalized patient records were reviewed after their discharge and the discharge diagnosis was entered as recorded per the clinician-in-charge. The WHO classical dengue case classification from 1997 was used for the diagnosis of DHF and dengue shock syndrome (DSS).

All CRFs were crosschecked for completeness by the medical research assistants and then passed on to the data management team at the central epidemiology office at the Ministry of Health in Colombo. A coding key was used and data entered in duplicate into a Microsoft SQL Server 2008 database by trained data-entry staff. Duplicate databases were compared and discrepancies resolved by referring to the original documents. The database was sent to Umeå University in Sweden for further data cleaning and random quality control.

### Laboratory testing

All laboratory testing was carried out at the Medical Research Institute which is the national center for laboratory services. A new dengue diagnostic laboratory at MRI was set up to process the samples for this study. Genetech Research Institute (GRI) Colombo, a private sector non-profit research institute was selected as a supporting reference laboratory to the project and functioned as an interim testing laboratory in the first year of the project to ensure smooth transition of diagnostic testing to the newly established MRI dengue laboratory.

Dengue screening RT-PCR was performed on all samples followed by serotyping by semi-nested PCR for all positive samples [10]. Dengue IgM capture ELISA (Standard Diagnostics, Korea) and Dengue NS1 antigen ELISA (Standard Diagnostics, Korea) were conducted on all samples according to manufacturer’s specifications. We defined current laboratory confirmed dengue diagnosis as PCR or NS1 positive. However, as a vast majority of patients were enrolled at day 4–5 of illness, when often PCR turns negative and dengue IgM positive, in order to capture all cases, we included dengue IgM positivity to the diagnosis of laboratory confirmed dengue. Results for PCR, NS1 and IgM positivity are reported separately and combined, as well as per day of illness

A sub-sample of sera from dengue positive and negative patients was sent to the Duke-NUS Graduate Medical School in Singapore once every 3 months for quality control assessments, virus isolation, and serotyping. Virus isolation was done using C6/36 Aedes albopictus cell lines and mosquito inoculation of selected samples[11]. Serotype-specific detection of dengue viruses was done by fourplex real-time reverse transcriptase PCR assay developed by the CDC[12]. To determine dengue specific IgG and dengue specific IgM we used an in-house protocol according to standard methodologies.

### Data analysis

Statistical analysis was performed with STATA version 12 (StataCorp, TX, USA). Sample proportions and means of demographic characteristics were presented in a descriptive table (Table 1). Categorical variables were compared by Fisher’s exact or Chi-square test, as appropriate, between groups. The Student’s t-test was used for continuous variables. Significance was assigned at *P* < 0.05 for all parameters and were two-sided unless otherwise indicated.

**Table 1:** Demographic characteristic of study population.

	Total (n = 3,127)	IPD (n = 2,160)	OPD (n = 964)	
Age (yrs)			Hospital (n = 667)	GP (n = 310)
• Mean (SD)	22.3 (17.5)	23.8 (16.8)	16.0 (17.6)	25.3 (18.9)
• Median	19	21	8	23
• Range	0–90	0–90	0–85	0–88
**Gender, n (%)**				
• Male	1737 (55.6)	1185 (54.9)	379 (56.8)	172 (55.5)
**Enrolment sites**	3127	2160 (69.1)	667 (21.3)	310 (9.90)
**Day of illness at first presentation (days)**				
• Mean(SD)	4.2 (1.57)	4.67 (1.38)	3.35 (1.40)	2.76 (1.54)
• Median	4	5	3	2
• Range	0–10	0–10	0–10	0–8
**DHF diagnosed at time of first presentation**	345 (11.0)	341 (15.8)	4 (0.6%)	0
**Outcome, n (%)**				
• Hospitalized	2230 (71.3)	2160 (100)	65 (9.75)	4 (1.35)
• Death	5 (0.16)	5 (100)	0	0
**Hospitalization duration (days)**^**#**^				
• Mean (SD)	4.13 (1.85)	3.58 (2.23)	0.20 (0.92)	-
• Median	4	4	0	
• Range	0–18	0–18	0–8	

## Results

### Demographic data

Between 1 April 2012 and 31 March 2014, we enrolled 3,127 patients of which 1737 (55.6%) were males. Of these 3,127 febrile cases, 90.1% (n = 2,817) were enrolled from the three sentinel hospitals, where 2,150 (76.3%) were from the inpatient departments (IPD), 667 cases (23.7%) from the hospitals outpatient departments (OPD) and; 310 patients (9.9%) were enrolled from the GPs (Table 1). The mean age of the study population was 22.3 years (SD 17.5; range 1 month to 90 years of age). The majority (80.6%) of the subjects were recruited (and hence blood samples collected) on day 3–6 of illness with a mean of 4.2 days (SD 1.6). The median day of illness at time of presentation to any of the sentinel sites was 4 for all cases, with the mean day of illness being 4.6 days for hospitalized cases (SD 1.40) and 3.1 (SD 1.46) for non-hospitalized patients (p<0.001). DHF cases presented on day 4.83 of illness (SD 1.33); lab confirmed dengue fever (without DHF) on day 4.69 (SD 1.31) and other febrile illnesses (OFI; all dengue assays negative) on day 4.22 (SD1.59). The average duration of hospitalization was 4.13 days (SD 1.85). Table 1 summarizes the differences between in-and outpatients in terms of demographic variables and outcomes. There were 5 deaths (0.16%), all classified by the clinicians as due to DHF, and all were laboratory confirmed dengue. The case fatality was 5 out of 525 DHF (0.95%).

### Laboratory diagnostics

**Fig 2:**
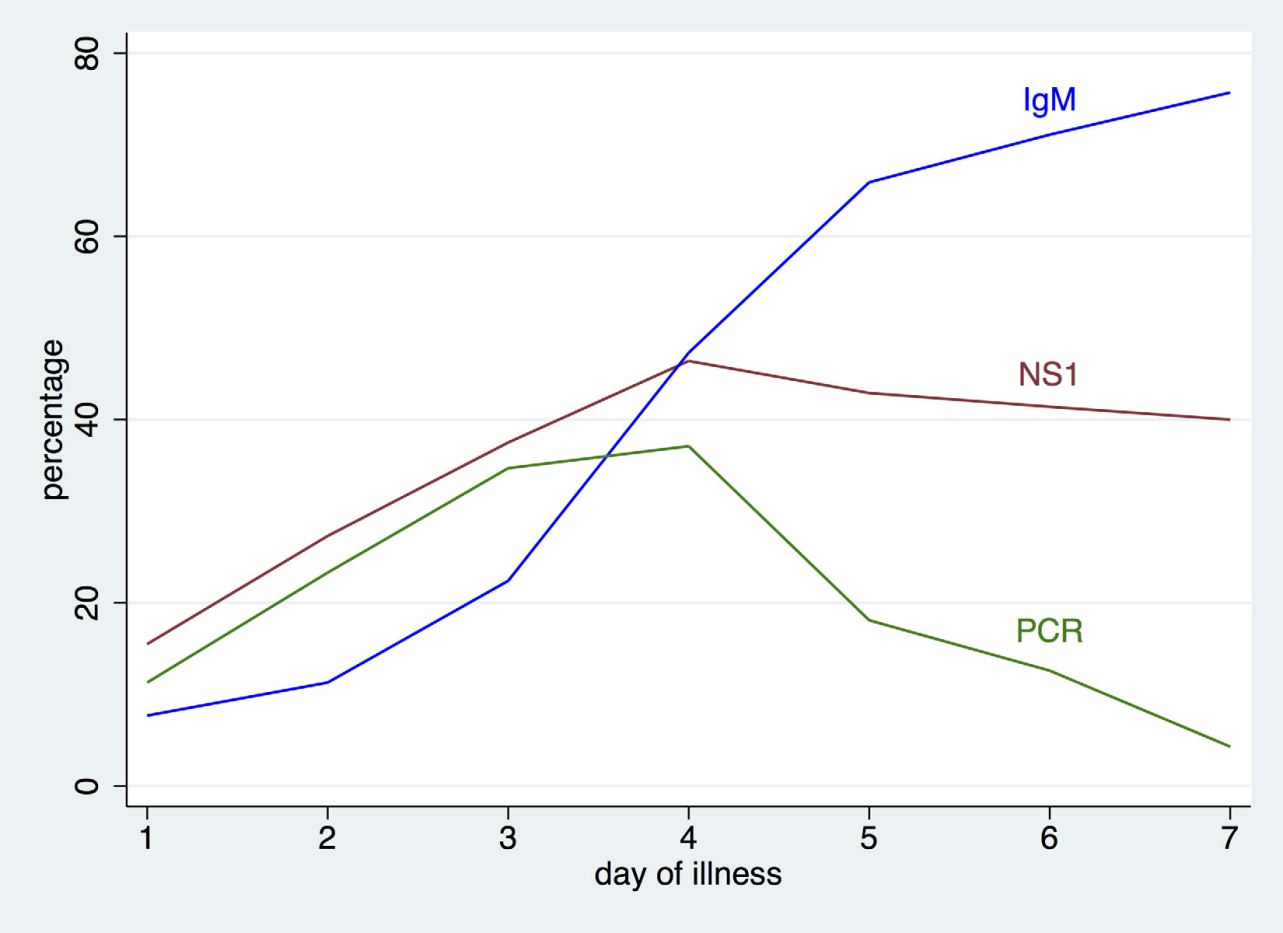
Percentage of dengue positive laboratory diagnostic test results out of 3,065 febrile cases by day of illness, using three different methods: dengue screening RT-PCR on all samples followed by semi-nested typing PCR for all positive samples (PCR); dengue IgM capture ELISA (Standard Diagnostics, Korea) (IgM) and dengue NS1 antigen ELISA (Standard Diagnostics, Korea) (NS1).

Of the 3,127 febrile cases, 43.6% were PCR and/or NS1 confirmed dengue cases (Table 2). Overall, the proportion of NS1 positivity was higher compared to PCR (Fig 2). The majority of the laboratory confirmed cases were from patients recruited from IPD (53.9%) compared to OPD (hospitals and GPs) (7.6%). Of patients presenting within 5 days of illness at GP settings, 36 out of 310 had a positive PCR or NS1 (11.6%). Table 2 shows the differences of the assay results between the inpatients and outpatients (hospital outpatients and GPs), both separately as well as in combination.

**Table 2:** Laboratory confirmation of dengue by RT-PCR, IgM ELISA or NS1 ELISA.

Laboratory confirmed dengue	n (%)			
Positive for either NS1, IgM or PCR	Total	IPD*	OPD* (Hospital)	OPD* (GP)
• Positive	1925 (61.6)	1687 (53.9)	182 (5.82)	55 (1.76)
• Negative	1189 (38.0)	466 (14.9)	483 (15.4)	238 (7.61)
Positive for IgM only	1633 (52.2)	1366 (43.7)	86 (2.75)	27 (0.86)
Positive for PCR and/or NS1 only	1363 (43.6)	1189 (38.0)	136 (4.35)	37 (1.18)
Positive for both IgM and NS1 only	856 (27.4)	813 (26.0)	34 (1.09)	8 (0.26)
Positive for both IgM and PCR only	404 (12.9)	375 (12.0)	25 (0.80)	4 (0.13)
Positive for IgM and NS1 and PCR	342 (10.9)	320 (10.2)	19 (0.61)	3 (0.10)
Laboratory results**				
• IgM-ELISA (+)	1480 (76.9)	1366 (71.0)	86 (4.47)	27 (0.86)
• NS1(+)	1247 (64.8)	1094 (56.8)	120 (6.23)	32 (1.66)
• PCR (+)	754 (39.2)	643 (33.4)	87 (4.52)	24 (1.25)

* percentages were calculated out of all febrile cases (n = 3127)

** These are calculated based on positive laboratory confirmed dengue cases, n = 1925 (ie. those with NS1, IgM or PCR positive)

#### Non-dengue cases (OFI = other febrile illnesses)

There were 1,189 cases (38%) for whom all dengue assays were negative (Table 2). The majority (23.1%) of those were outpatients (hospital and GP) compared to 466 (14.9%) in the inpatients. The majority of the non-dengue hospitalized cases were recorded by the clinicians as ‘viral fevers’ or respiratory infections at discharge.

#### Dengue assays by day of illness

We looked at the proportion of positive assays by day of illness. If presenting within the first–three days after onset of illness, a large proportion of febrile cases were not dengue. The longer the duration of fever, the more likely the patient had laboratory confirmed dengue (Fig 2). IgM displayed an increasing positivity with day of illness; the proportion of patients with a positive PCR decreased after peaking at day 4 of illness. Fig 2 also shows that NS1 reflects the PCR positivity curve, but is higher than PCR and remains positive longer than PCR.

### Serotypes, quality assurance, and secondary infections

A subset of samples (n = 536) was sent to the Emerging Infectious Diseases Program Laboratory at the Duke-NUS Graduate Medical School in Singapore for serotyping, quality assurance (IgM and PCR) and secondary infections (dengue IgG). Serotyping identified DENV-1 in 85% and DENV-4 in 15%. The percentage agreement for both PCR and IgM tests between the Singapore and Sri Lanka was 80.7% and 77.5%, respectively (Table 3). Using positive virus isolation as gold standard, the sensitivity and specificity of the Sri Lanka performed PCR was similar to the Singapore performed PCR, confirming the quality of the Sri Lanka laboratory (Table 4).

**Table 3:** Comparison between IgM and PCR assays from DUKE NUS and Sri Lanka.

	Both positive	Both Negative	Total tested	% agreement
IgM	148	263	530	77.5
PCR	298	120	518	80.7

**Table 4:** Comparison of PCR and NS1 sensitivity and specificity against virus isolation for samples from DUKE NUS and Sri Lanka.

	SG PCR (%)	SL PCR (%)	SL NS1 (%)
Sensitivity (95% CI)	87.4 (82.9–91.0)	98.6 (96.5–99.6)	93.7 (90.2–96.2)
Specificity (95% CI)	61.8 (55.4–68.0)	62.9 (56.7–68.9)	61.0 (54.6–67.0)

SL PCR = Sri Lanka PCR

SL NS1 = Sri Lanka NS1

SG PCR = Singapore (DUKE-NUS) PCR

SG virus isolation = Singapore (DUKE-NUS) virus isolation

Based on IgG testing in the subset of samples at Duke-NUS, the overall IgG seroprevalence (in patients with or without dengue) was 74.4% with increasing percentages from 67.9% in those below the age of 18 to 90.3% in those above age 45. In the absence of a paired sample, we used a positive dengue specific IgG < 7 days as proxy for a secondary dengue infection. The proportion of patients with PCR or NS1 confirmed dengue who had secondary dengue infections (positive IgG) was 57.1%. NS1 and PCR positivity was higher in patients with primary infections (defined as IgG negative) (96% and 98%) compared to secondary infections (IgG positive) (81.2% and 83.7%) while IgM positivity was higher in patients with secondary infections (60.2%) in the selected sample set sent to Duke-NUS. As we only did dengue IgG in a sub-set of patients, we were not able to correlate secondary versus primary infections with disease outcome in the total set of patients.

### Clinical diagnosis

Medical research assistants reviewed the signs and symptoms of all cases and made a presumptive clinical diagnosis at first presentation to the sentinel sites. Among cases that were clinically classified as DF or DHF (n = 2081), 1653 (79.4%) were laboratory confirmed dengue. The majority of the cases diagnosed at time of interview as DF and DHF cases were from IPD (92.9%, n = 1934) compared to OPD (7.02%, n = 146). In other words, dengue was over-diagnosed in the in-patient setting and under-diagnosed in outpatient settings. Those who presented with a clinical diagnosis of DHF had a far higher proportion of being laboratory dengue confirmed than those with a presumptive diagnosis of dengue fever (93.6% versus 76.6%). Using the definition of any of the 3 diagnostics assay (NS1, PCR or IgM) as positive for ‘laboratory confirmed’ dengue, the sensitivity and specificity of the trained medical research assistant in distinguishing dengue (includes both DHF and DF cases) from OFI at the time of interview was 87.3% and 64.3% respectively (Table 5). The sensitivity was higher for IPD patients (96.1%) compared to OPD (25.9%). The positive and negative predictive values are presented in Table 5. The presumptive clinical diagnosis of the clinicians-in-charge (who were not part of the study) was obtained from the source files (clinical records). These diagnoses were only available for hospitalized cases (n = 2,230). Using laboratory confirmed dengue as the ‘gold standard’, the sensitivity of the presumptive clinical diagnosis for dengue vs OFI at the time of first contact was 84.7%, versus 94.2% at the time of discharge (Table 5). However the specificity was low for both: 32.5% at admission and 37.1% at discharge.

**Table 5:** Sensitivity, specificity, positive and negative predictive values of clinical diagnosis in distinguishing Dengue (DHF+DF) from Other Febrile Illness (OFI).

**For all patients (at time of interview) by trained clinical research assistant**			
**Dengue vs OFI**	% (95% CI)		
	Total	IPD	OPD
• Sensitivity	87.3 (85.7–88.8)	96.1 (95.1–97.0)	(20.5–32.1)
• Specificity	64.3 (61.5–67.0)	27.4 (23.4–31.7)	(85.6–90.5)
• Positive Predictive Value	79.6 (77.8–81.3)	82.5 (80.7–84.1)	(33.9–50.5)
• Negative Predictive Value	76.1 (73.3–78.7)	66.5 (59.3–73.1)	78.3 (75.3–81.1)
**For hospitalized patients only, by routine clinician-in-charge**			
**Dengue vs OFI**	% (95% CI)		
•	At admission		At discharge
• Sensitivity	84.7 (82.9–86.4)		(92.9–95.2)
• Specificity	32.5 (28.2–36.9)		(32.7–41.8)
• Positive Predictive Value	82.3 (80.5–84.1)		(83.2–86.5)
• Negative Predictive Value	36.4 (31.8–41.3)		62.8 (56.8–68.6)

**NOTE**: For Dengue vs OFI, the sensitivity of diagnosis is against the laboratory confirmed dengue cases defined as positive for either NS1, PCR or IgM.

**Fig 3:**
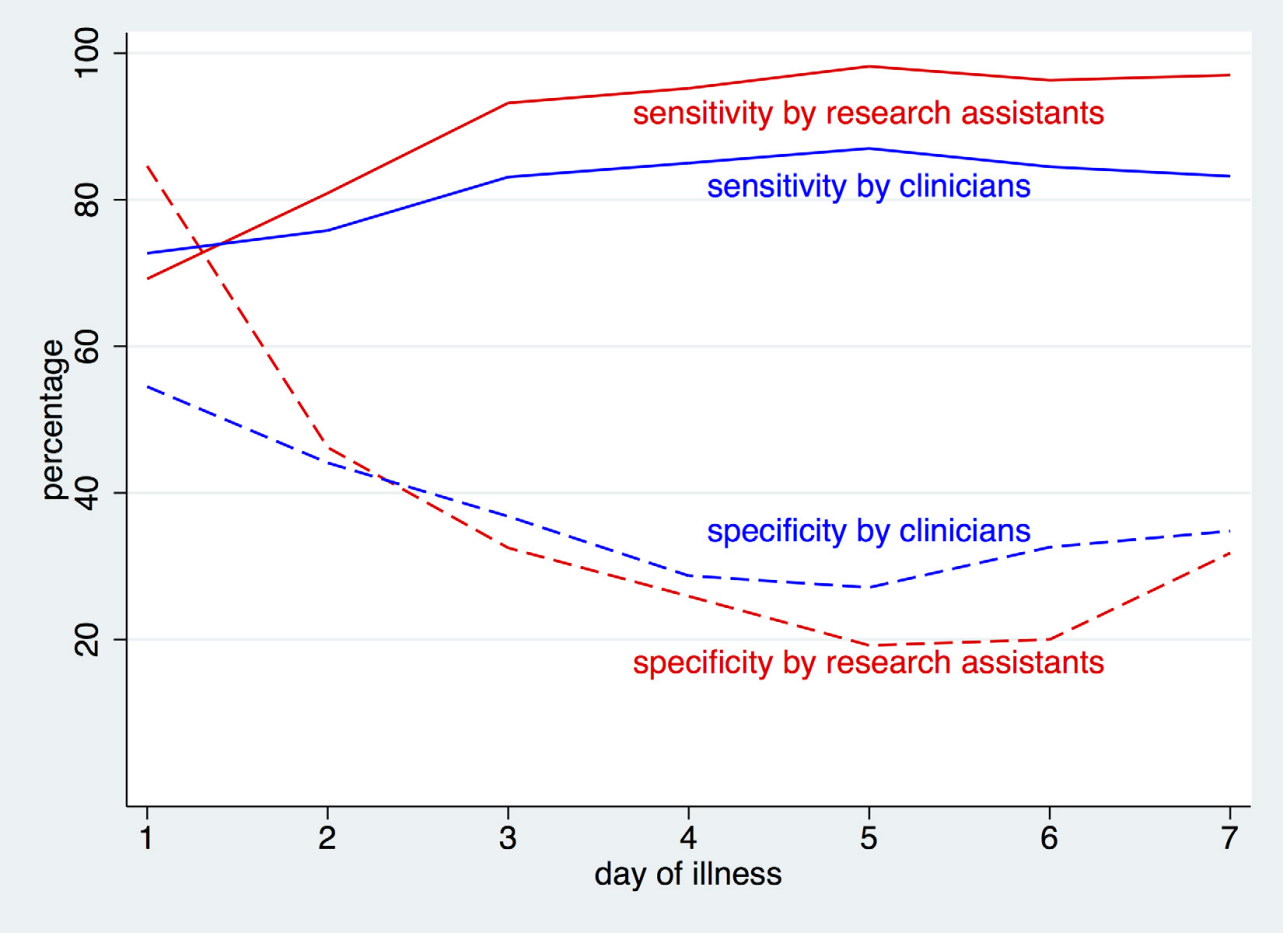
Sensitivity and specificity for distinguishing between dengue fever and other febrile illnesses by day of illness, as diagnosed by research assistants (2,142 hospitalised patients) and clinicians (2,136 hospitalised patients).

Fig 3 shows the changes of sensitivity and specificity per day of illness at first presentation and compares the sensitivity and specificity of the routine clinicians-in charge versus the trained research assistant. Sensitivity increases with the duration of illness, while specificity remains low throughout. The sensitivity of the trained research assistant was higher than that of the clinicians-in-charge, while the specificity was higher for the clinicians-in-charge.

## Discussion

Syndromic surveillance is the mainstay of many surveillance systems in dengue endemic countries including Sri Lanka, but is known to lack sensitivity and specificity[4]. A laboratory-enhanced sentinel surveillance system provides more precise information to public health authorities and policy-makers on virus serotype and disease severity (as measured as proportion of DHF over all dengue cases), in addition to location and time. Here we report the results of 2 years of laboratory-enhanced sentinel surveillance in the Colombo District of Sri Lanka. Several important observations can be drawn.

First, we showed that a large proportion of febrile illnesses (46.3%) in the sentinel surveillance sites were in fact due to dengue infections, thus highlighting the burden of dengue in Sri Lanka. The proportion of dengue as a cause of febrile illness was–as expected -far higher in cases seen in the hospitals versus GPs. Because the majority of cases enrolled in this surveillance were from hospitals, the proportion of lab confirmed dengue in this study is higher than that reported from other surveillance systems which usually report between 5–15%[4]. The proportion of dengue seen at GP settings (11.6%) is consistent with the latter. The high dengue burden that we documented was consistent with the peak of dengue cases reported to the Ministry of Health in the same time period[9]. The mean age in our study was 22 years highlighting that adults are also affected by dengue in Sri Lanka. The mean duration of hospitalization was 4.1 days; the frequent hospitalization of dengue cases and the length of around 4 days contribute appreciably to the economic burden of dengue in endemic countries.

Second, we were able to document the specific serotypes responsible for dengue infections in 2012–2014. All laboratory confirmed cases were due to either DENV-1 or DENV-4, with DENV-1 being the predominant serotype (85%). DENV-1 replaced DENV-3 in 2009 triggering a wave of severe dengue epidemic in Sri Lanka, all associated with a higher incidence and mortality than with any previously recorded epidemic in the country [13]. Bayesian phylogeographic analyses suggest that the 2009 Sri Lankan epidemic DENV-1 strain may have been imported from Thailand, then spread within Sri Lankan, and from there it spread further to Pakistan and Singapore[13]. Sustained outbreaks over several years due to one serotype are unusual. Because of herd immunity, most epidemics usually only last for about one year and subsequent outbreaks are then triggered by the introduction of a new serotype[3, 14, 15]. However, genetic changes in the virus over time also within a country can occur independent of new introductions from other countries thereby triggering new outbreaks [16]. For example, in 1989 the existing genotype of DENV-3 in Sri Lanka was shown to have undergone a lineage change which led to the first dengue hemorrhagic fever outbreak despite the fact that this serotype had been in circulation since at least 1981[17]. Sequencing of the DENV-1 taken from different time periods is still ongoing, the results of which may help us understand why this serotype was associated with sustained epidemic transmission for 6 years.

Third, we documented a high proportion of DHF. In Sri Lanka, the 1997 WHO dengue case classification is still used routinely by clinicians, hence we relied on the managing clinicians’ diagnosis using the classification they are familiar with rather than the 2009 TDR dengue case classification. Dengue hemorrhagic fever (DHF) was diagnosed in 11% of patients at the time of first contact. For hospitalized cases, 22% were discharged with the diagnosis of DHF (with 5 fatal outcomes due to DHF). A DHF proportion of 22% is unusually high, as most studies report a proportion of 5–10% underscoring that the current epidemic is associated with more severe disease. Patients who had signs and symptoms of DHF already at first encounter to our sentinel system presented much later compared to dengue fever or OFI, which could be one explanation why such cases have a worse outcome. 0.9% of the DHF cases died.

Fourth, to assess whether costly diagnostic assays are indeed worthwhile in a sentinel surveillance system, we compared the clinicians’ presumptive clinical diagnoses with the laboratory confirmation. The clinicians’ diagnosis for dengue at time of admission had a sensitivity of 84.7% and specificity of 32.5%. The positive predictive value for the clinicians was 82.3% and the negative predictive value 36.4% at time of admission. The sensitivity and specificity was higher at discharge, most likely because the clinicians by then had all laboratory tests at hand and were familiar with the clinical evolution over time, even without knowing the results of our assays. The trained research assistants had a higher sensitivity and specificity compared to the clinicians who saw the patients as part of their daily clinical routine on busy days. The higher sensitivity by the research assistants who were all medically trained can be explained by the fact that they spent more time reviewing all the symptoms and signs as documented by our standardized case report form. This underscores that training does indeed improve sensitivity. However, having said this, the sensitivity for the routine clinicians-in-charge was still high, as Sri Lankan doctors are very familiar with dengue. Furthermore, we were able to show that the sensitivity and specificity depends on the day of illness (Fig 3), with the highest sensitivity and specificity later in the course of illness. In other words, if patients presented later (after day 3–4 of illness), sensitivity increased, while specificity remained low. Mild respiratory illnesses and non-specific viral fevers are usually of shorter duration and hence present a large proportion of febrile patients seen in the first 3 days of illness. Patients with DHF were much more often correctly diagnosed as a laboratory confirmed dengue case. This finding is consistent with previous reports from Thailand, where the authors concluded that clinicians had a 62% sensitivity and 92% specificity in identifying DHF according to the WHO’s definition, without the need for laboratory confirmation of dengue virus infection in endemic areas[18]. DHF presents clinically with a very characteristic constellation of clinical symptoms, signs and changes in leukocytes, platelets, and hematocrit; a constellation that is so pathognomonic for DHF that WHO in its 2009 revised dengue case classification does not require laboratory confirmation for severe disease but does require laboratory confirmation for dengue with or without warning signs) [19]. Overall, our findings show that the clinicians’ suspicion of dengue was very high as seen in the high sensitivity, but specificity was very low. To enhance specificity it is important to add laboratory confirmation of dengue.

Fifth, we were able to assess three diagnostic essays (PCR, Dengue IgM ELISA and NS1 ELISA) in a sentinel surveillance setting. For dengue, day of illness determines the choice of diagnostic assay. During the viremic phase (up to day 4–5 of illness), molecular biological or virological approaches such as PCR or NS1 should be employed; after the viremic phase (≥5 days), serological assays are indicated, with dengue IgM being the most frequently used assay [20]. Our figures confirm the temporal changes in positive assays per day of illness, with PCR and NS1 being positive in the earlier phase, and IgM later. A combination of methods that target different time periods maximizes diagnostic sensitivity. Given the constraints of such a large sentinel surveillance as ours, it was programmatically not feasible to take a convalescent serum at 14–21 days after discharge which would have helped in confirming the diagnosis–hence we lack a definitive “gold standard” diagnosis in those patients where we only have a single IgM result. In the absence of convalescent sera, we defined “dengue IgM, NS1 or PCR positive” as laboratory confirmed dengue which may have overestimated the results, as some IgM positive (if NS1 or PCR negative) cases may have been recent dengue infections rather than current (IgM can remain positive for 3 months). Hence, we also report our results for PCR, NS1 and IgM separately and in different combinations. The proportion of PCR and/or NS1 positive of all 3,127 febrile cases was 43.6%, but the proportion of PCR and/or NS1 and/or IgM was 61.6%. As NS1 based assays are increasingly used in endemic countries, we also particularly looked at the issues of NS1 as measured by ELISA. NS1 was positive for more days of illness compared with PCR. Indeed, NS1 can be found in the peripheral blood circulation for up to 9 days from illness onset, but can persist for up to 18 days for some cases[20, 21]. Hence NS1 offers a larger window of opportunity for diagnosis of dengue compared with virus isolation and PCR[20]. Furthermore, the proportion of NS1 positive subjects in the first 5 days of illness was higher than that of PCR. Higher sensitivity of NS1 compared with PCR has been documented in some studies [22–24] but not in others[25]. It is important to note that we tested NS1 by ELISA and not with the cheaper rapid diagnostic (RDT) kits that are now widely available. Hunsperger et al showed that sensitivity of NS1 by ELISA is higher (60–75%) compared with NS1 RDT (38–71%) [24]. Hunsperger’s analysis also showed that NS1 was more sensitive in primary versus secondary infections, an observation that we can confirm with our findings.

Six, with the EU funded laboratory-enhanced surveillance project a dedicated government laboratory was set up to perform routine molecular testing for dengue. As a result, the capacity to do PCR and serotyping is now well established in Colombo. Quality assurance with a subset of samples sent to the Duke-NUS Laboratory “Emerging Infectious Diseases Program” showed a high agreement. At Duke-NUS, we also did virus isolation. Although virus isolation is highly specific, the sensitivity is reported to be only approximately 40% [26]. Virus isolation has the advantage of providing a virus isolate that can be used for further genome sequencing, or virus neutralization and other in vitro studies[20], but it requires highly trained operators, depends on a short viremia period, thus providing only a narrow window of opportunity from illness onset; in quintessence, it is not a diagnostic approach suitable for developing countries. Cost effectiveness studies are needed to evaluate the need for assays such as PCR on a routine basis in Sri Lanka compared to the more affordable NS1 only; and our sentinel surveillance can potentially serve as a basis for such studies.

The advantage of laboratory enhanced sentinel surveillance is that it is less expensive (being restricted to small areas) and produces data of higher quality than nationwide passive syndromic surveillance. The disadvantage of sentinel surveillance however is the inability to ensure that the sample population is representative and the inability to calculate incidence rates compared to cohort studies. The age distribution in our cohort for example is largely representative of the hospitals selected, and the high proportion of laboratory confirmed dengue reflects the fact that the majority of subjects were recruited from hospitals.

In conclusion, dengue poses a high burden in Sri Lanka as evidenced by a substantial proportion of laboratory confirmed dengue cases in our sentinel surveillance system. DENV-1 and to a lesser degree, DENV-4 infection were responsible for a high proportion of febrile illnesses in Colombo during the years 2012–2014. Clinicians’ diagnoses were associated with high sensitivity, but laboratory confirmation is required to enhance specificity. Adding laboratory confirmation to syndromic surveillance will add costs, but laboratory confirmation will also enhance specificity, help monitor changes in serotype distribution, new serotype introduction, and help to better define trigger thresholds for intensified vector control measures.

## References

[1] Murray NE, Quam MB, Wilder-Smith A. Epidemiology of dengue: past, present and future prospects. Clin Epidemiol. 2013;5:299–309. Epub 2013/08/31. 10.2147/CLEP.S34440 23990732 PMC3753061

[2] Focks DA, Daniels E, Haile DG, Keesling JE. A simulation model of the epidemiology of urban dengue fever: literature analysis, model development, preliminary validation, and samples of simulation results. Am J Trop Med Hyg. 1995;53(5):489–506. Epub 1995/11/01. . 7485707 10.4269/ajtmh.1995.53.489

[3] Wilder-Smith A, Ooi EE, Vasudevan SG, Gubler DJ. Update on dengue: epidemiology, virus evolution, antiviral drugs, and vaccine development. Curr Infect Dis Rep. 2010;12(3):157–64. Epub 2011/02/11. 10.1007/s11908-010-0102-7 . 21308524

[4] Runge-Ranzinger S, Horstick O, Marx M, Kroeger A. What does dengue disease surveillance contribute to predicting and detecting outbreaks and describing trends? Trop Med Int Health. 2008;13(8):1022–41. Epub 2008/09/05. doi: TMI2112 [pii] 10.1111/j.1365-3156.2008.02112.x . 18768080

[5] Jaenisch T, Idams, Sakuntabhai A, Denfree, Wilder-Smith A, DengueTools. Dengue research funded by the European commission-scientific strategies of three European dengue research consortia. PLoS neglected tropical diseases. 2013;7(12):e2320 Epub 2013/12/19. 10.1371/journal.pntd.0002320 24349584 PMC3861113

[6] Wilder-Smith A, Renhorn KE, Tissera H, Abu Bakar S, Alphey L, Kittayapong P, et al DengueTools: innovative tools and strategies for the surveillance and control of dengue. Global health action. 2012;5 Epub 2012/03/28. 10.3402/gha.v5i0.17273 22451836 PMC3312611

[7] Kanakaratne N, Wahala WM, Messer WB, Tissera HA, Shahani A, Abeysinghe N, et al Severe dengue epidemics in Sri Lanka, 2003–2006. Emerg Infect Dis. 2009;15(2):192–9. Epub 2009/02/06. 19193262 10.3201/eid1502.080926 PMC2662655

[8] Tissera HA, Ooi EE, Gubler DJ, Tan Y, Logendra B, Wahala WM, et al New dengue virus type 1 genotype in Colombo, Sri Lanka. Emerg Infect Dis. 2011;17(11):2053–5. 10.3201/eid1711.101893 22099096 PMC3310553

[9] Disease Surveillance: Trends Sri Lanka Epidemiology Unit Ministry of Health Sri Lanka [cited 2015]. Available from: http://www.epid.gov.lk/web/index.php?option=com_casesanddeaths&Itemid=448&lang=en.

[10] Lanciotti RS, Calisher CH, Gubler DJ, Chang GJ, Vorndam AV. Rapid detection and typing of dengue viruses from clinical samples by using reverse transcriptase-polymerase chain reaction. J Clin Microbiol. 1992;30(3):545–51. . 1372617 10.1128/jcm.30.3.545-551.1992 PMC265106

[11] Choy MM, Ellis BR, Ellis EM, Gubler DJ. Comparison of the mosquito inoculation technique and quantitative real time polymerase chain reaction to measure dengue virus concentration. Am J Trop Med Hyg. 2013;89(5):1001–5. 10.4269/ajtmh.13-0100 24019432 PMC3820311

[12] Gurukumar KR, Priyadarshini D, Patil JA, Bhagat A, Singh A, Shah PS, et al Development of real time PCR for detection and quantitation of Dengue Viruses. Virology journal. 2009;6:10 10.1186/1743-422X-6-10 19166574 PMC2651855

[13] Ocwieja KE, Fernando AN, Sherrill-Mix S, Sundararaman SA, Tennekoon RN, Tippalagama R, et al Phylogeography and molecular epidemiology of an epidemic strain of dengue virus type 1 in Sri Lanka. Am J Trop Med Hyg. 2014;91(2):225–34. 10.4269/ajtmh.13-0523 24799375 PMC4125241

[14] Bennett SN, Holmes EC, Chirivella M, Rodriguez DM, Beltran M, Vorndam V, et al Molecular evolution of dengue 2 virus in Puerto Rico: positive selection in the viral envelope accompanies clade reintroduction. J Gen Virol. 2006;87(Pt 4):885–93. Epub 2006/03/11. doi: 87/4/885 [pii] 10.1099/vir.0.81309–0 . 16528038

[15] Rigau-Perez JG, Vorndam AV, Clark GG. The dengue and dengue hemorrhagic fever epidemic in Puerto Rico, 1994–1995. Am J Trop Med Hyg. 2001;64(1–2):67–74. Epub 2001/06/27. . 11425166 10.4269/ajtmh.2001.64.67

[16] Bennett SN, Holmes EC, Chirivella M, Rodriguez DM, Beltran M, Vorndam V, et al Selection-driven evolution of emergent dengue virus. Molecular biology and evolution. 2003;20(10):1650–8. 10.1093/molbev/msg182 . 12832629

[17] Messer WB, Gubler DJ, Harris E, Sivananthan K, de Silva AM. Emergence and global spread of a dengue serotype 3, subtype III virus. Emerg Infect Dis. 2003;9(7):800–9. 12899133 10.3201/eid0907.030038 PMC3023445

[18] Srikiatkhachorn A, Gibbons RV, Green S, Libraty DH, Thomas SJ, Endy TP, et al Dengue hemorrhagic fever: the sensitivity and specificity of the world health organization definition for identification of severe cases of dengue in Thailand, 1994–2005. Clinical infectious diseases: an official publication of the Infectious Diseases Society of America. 2010;50(8):1135–43. 10.1086/651268 20205587 PMC2853952

[19] Dengue: guidelines for diagnosis, treatment, prevention and control Organization WH, editor. Geneva: World Health Organization; 2009. 23762963

[20] Tang KF, Ooi EE. Diagnosis of dengue: an update. Expert review of anti-infective therapy. 2012;10(8):895–907. 10.1586/eri.12.76 . 23030329

[21] Peeling RW, Artsob H, Pelegrino JL, Buchy P, Cardosa MJ, Devi S, et al Evaluation of diagnostic tests: dengue. Nature reviews Microbiology. 2010;8(12 Suppl):S30–8. Epub 2011/05/07. . 21548185 10.1038/nrmicro2459

[22] Chen X, Chen R, Gu W, He J, Cai W, Li J, et al Clinical evaluation of dengue RNA, NS1, and IgM for diagnosis of dengue in Southern China. Journal of medical virology. 2015 10.1002/jmv.24314 . 26118588

[23] Pok KY, Lai YL, Sng J, Ng LC. Evaluation of nonstructural 1 antigen assays for the diagnosis and surveillance of dengue in Singapore. Vector borne and zoonotic diseases. 2010;10(10):1009–16. 10.1089/vbz.2008.0176 20426686 PMC2992696

[24] Hunsperger EA, Yoksan S, Buchy P, Nguyen VC, Sekaran SD, Enria DA, et al Evaluation of commercially available diagnostic tests for the detection of dengue virus NS1 antigen and anti-dengue virus IgM antibody. PLoS neglected tropical diseases. 2014;8(10):e3171 10.1371/journal.pntd.0003171 25330157 PMC4199549

[25] Ahmed NH, Broor S. Comparison of NS1 antigen detection ELISA, real time RT-PCR and virus isolation for rapid diagnosis of dengue infection in acute phase. Journal of vector borne diseases. 2014;51(3):194–9. . 25253212

[26] Chua KB, Mustafa B, Abdul Wahab AH, Chem YK, Khairul AH, Kumarasamy V, et al A comparative evaluation of dengue diagnostic tests based on single-acute serum samples for laboratory confirmation of acute dengue. The Malaysian journal of pathology. 2011;33(1):13–20. . 21874746

